# Neural correlates of sensorimotor gating: a metabolic positron emission tomography study in awake rats

**DOI:** 10.3389/fnbeh.2014.00178

**Published:** 2014-05-22

**Authors:** Cathrin Rohleder, Fabienne Jung, Hanna Mertgens, Dirk Wiedermann, Michael Sué, Bernd Neumaier, Rudolf Graf, F. Markus Leweke, Heike Endepols

**Affiliations:** ^1^Department of Psychiatry and Psychotherapy, Central Institute of Mental Health, Medical Faculty Mannheim, Heidelberg UniversityMannheim, Germany; ^2^Multimodal Imaging, Max Planck Institute for Neurological ResearchCologne, Germany

**Keywords:** prepulse inhibition, startle, FDG-PET, glucose utilization, translational research, neuropsychiatric disorders, neural network

## Abstract

Impaired sensorimotor gating occurs in neuropsychiatric disorders such as schizophrenia and can be measured using the prepulse inhibition (PPI) paradigm of the acoustic startle response. This assay is frequently used to validate animal models of neuropsychiatric disorders and to explore the therapeutic potential of new drugs. The underlying neural network of PPI has been extensively studied with invasive methods and genetic modifications. However, its relevance for healthy untreated animals and the functional interplay between startle- and PPI-related areas during a PPI session is so far unknown. Therefore, we studied awake rats in a PPI paradigm, startle control and background noise control, combined with behavioral [^18^F]fluoro-2-deoxyglucose positron emission tomography (FDG-PET). Subtractive analyses between conditions were used to identify brain regions involved in startle and PPI processing in well-hearing Black hooded rats. For correlative analysis with regard to the amount of PPI we also included hearing-impaired Lister hooded rats that startled more often, because their hearing threshold was just below the lowest prepulses. Metabolic imaging showed that the brain areas proposed for startle and PPI mediation are active during PPI paradigms in healthy untreated rats. More importantly, we show for the first time that the whole PPI modulation network is active during “passive” PPI sessions, where no selective attention to prepulse or startle stimulus is required. We conclude that this reflects ongoing monitoring of stimulus significance and constant adjustment of sensorimotor gating.

## Introduction

One of the most powerful paradigms in the field of schizophrenia psychophysiology is the prepulse inhibition (PPI) of the startle reflex (Swerdlow et al., 2008). PPI implies reduction of the startle reflex when a non-startling stimulus (prepulse) is presented 20–800 ms (Hoffman and Ison, 1980) before the startling stimulus. It is thought that PPI reflects protection of ongoing processing of the prepulse from interference by the startling pulse (Graham, 1975; Koch, 1999). Hence, PPI is used as a measure of sensorimotor gating, i.e., the ability of a weak stimulus to control (gate) the reflexive motor response to a subsequent intense stimulus. Apart from schizophrenia (Braff et al., 1978; Parwani et al., 2000; Swerdlow et al., 2006), PPI impairments are found in obsessive compulsive disorder (Ahmari et al., 2012), Gilles de la Tourette's syndrome (Swerdlow et al., 2001b), Huntington's (Swerdlow et al., 1995), Parkinson's (Valls-Sole et al., 2004) and Alzheimer's disease (Ueki et al., 2006). Compromised PPI is therefore regarded as an endophenotype for neuropsychiatric disorders and is widely used to characterize new animal models and antipsychotic potential of new drugs (Braff et al., 2001; Koch and Fendt, 2003; Swerdlow et al., 2008).

So far, invasive methods such as brain lesions, substance administration, electrical stimulation (Swerdlow et al., 2001a) and genetic approaches like selective breeding, inbred strain comparisons and mutant analysis (Swerdlow et al., 2000; Geyer et al., 2002; Schwabe et al., 2007) served to study brain circuitries regulating startle and PPI. The proposed pathway mediating PPI of acoustic startle comprises inferior and superior colliculus, pedunculopontine and laterodorsal tegmental nuclei as well as substantia nigra. Activated by a prepulse, the tegmental areas attenuate activity of the caudal pontine nucleus, which is a sensorimotor interface area of the startle reflex pathway. Besides, PPI seems to be modulated by a circuit including nucleus accumbens, ventral pallidum, septohippocampal system, basolateral amygdala, mediodorsal thalamus, and medial prefrontal cortex. It is assumed that this modulation pathway reduces sensorimotor gating by its link via nucleus accumbens and ventral pallidum to the mediation pathway (Koch and Fendt, 2003).

Invasive studies undoubtedly elucidate the general significance of a brain area for a given behavior. However, interventions alter the functionality of the neural network, which may conceal the true relevance of a lesioned brain area. The importance of a brain region may be underestimated because other regions are able to compensate. Or it may be overestimated because it is a relay station rather than a processing unit. The next step to understand startle and PPI at a systems level is therefore to validate the proposed network in intact organisms, and to allocate brain areas to different aspects of this behavior. Recently, functional magnetic resonance imaging (fMRI) has been used to investigate PPI in healthy humans (Goldman et al., 2006; Campbell et al., 2007; Kumari et al., 2008) and schizophrenia patients (Kumari et al., 2007). However, it is difficult to analyze PPI with fMRI in awake animals, because inevitable movements during startle preclude simultaneous scanning. We here combined a standard (passive) PPI paradigm, which does not require attention, with [^18^F]fluoro-2-deoxyglucose positron emission tomography (FDG-PET) to investigate functional metabolic brain activity during startle and PPI-mediation in awake rats. During the behavioral task FDG accumulates in active brain cells and can be visualized by a subsequent scan under anesthesia, enabling functional analysis of startle and PPI processing in small animals. This behavioral PET approach combines the advantages of [^14^C]-2-deoxyglucose autoradiography on the one hand, where animals can move freely during the uptake period but have to be sacrificed afterwards, and fMRI on the other hand, where one animal can be measured several times but its head has to remain fixed in the gantry.

## Materials and methods

### Subjects

In total 19 healthy, untreated male adult (postnatal day >106, 388 ± 48 g) rats were used. Fourteen of them were Black hooded rats (Janvier, France), five were Lister hooded (Charles River, Germany). Both originate from the same rat strain of the Lister Institute, but Lister hooded rats derived from outbred, Black hooded from inbred breeding. Animals were housed in pairs in type 4 cages enriched with a nest box and a horizontal tube for climbing in a temperature- and humidity-controlled room (22 ± 1°C, 55 ± 5% rh) on an inversed 12-h light/dark schedule (lights on 8:30 p.m–8:30 a.m.). They had free access to water, but diet was restricted. Experiments were conducted according to the German law on animal protection and approved by the local animal care committee.

All experiments took place during the dark, i.e., active phase of the rats' day-night cycle.

### Hearing test: brainstem auditory evoked potentials (BAEPs)

After an initial anesthesia (5% isoflurane in O_2_/N_2_O (3:7)) rats were transferred into a sound-attenuated chamber surrounded by a Faraday cage. Inhalation anesthesia was sustained during the whole procedure (2% isoflurane) and body temperature was maintained at 37°C. Click signals (1–32 kHz, 50 μs, presentation rate: 4 Hz, stimulus polarity: rarefraction; SigGen software, Tucker Davis Technology®, Alachua, USA) were presented via plastic tubes connected to closed-field speakers (TDT) that were inserted into the outer ear channel of the rats. The hearing test was conducted unilaterally, and started at a sound pressure level of 100 dB pSPL LIN, which was then reduced in steps of 10 dB pSPL LIN until no further response was elicited. The contralateral ear was masked by continuous white noise (30 dB pSPL LIN below stimulus level).

BAEPs were recorded at a rate of 20 kHz using two-channel difference recording with platinum-needle electrodes inserted subcutaneously behind left or right ear vs. vertex. The ground electrode was placed at the animals' back. Signals were amplified (×1000), low-pass filtered (cut-off: 3 kHz) and digitized before post-processing, which comprised further amplification (×1000) and high-pass-filtering (cut-off: 100 Hz) using Dasylab (National Instruments®, Austin, TX, USA). Thereafter, 1000 sweeps were averaged to visualize BAEPs. Rats' hearing threshold was determined as the average of the lowest sound pressure level that elicited BAEPs and the successive descending stimulus intensity, which induces no detectable response. Peak sound pressure level (dB pSPL LIN) was converted to continuous sound pressure level (dB SPL LIN) by means of a correction factor (=0.8509).

### Prepulse inhibition of acoustic startle reflex

The PPI paradigm was executed using SR-Lab (San Diego Instruments®, San Diego, USA). All presented acoustic stimuli consisted of white noise (frequency range: 2.2–16.7 kHz). The duration of all stimuli (startle as well as prepulses) was 25 ms. All trials were presented with an randomized interstimulus interval of 1–13 s. The background noise level was kept at 65 dB SPL LIN. An experimental session took 45 min and started with a habituation program, consisting of 1 min background noise and subsequently 25 initial startle trials with a sound pressure level of 110 dB SPL LIN. Habituation was followed by the actual PPI paradigm, consisting of 300 trials presented in a pseudorandomized order. Those trials included: (a) 30 control trials (background noise), (b) 30 startle-alone trials (110 dB SPL LIN), (c) 120 prepulse-alone trials (30 for each sound pressure level: 68, 72, 78, or 84 dB SPL LIN) and (d) 120 PPI trials where startle pulses were preceded by 68, 72, 78, or 84 dB SPL LIN prepulses (30 for each sound pressure level). The interval between prepulse and startle was 100 ms. After 20 further startle trials, the session was finished and the enclosure was cleaned with diluted acetic acid.

Startle amplitude was measured as integrated response over 100 ms, starting 5 ms after startle stimulus onset (recording range: 5–105 ms). All values were baseline corrected. To allow for a comparison of the absolute startle-alone amplitudes, those were normalized to daily calibration values. PPI was calculated for each prepulse intensity as percent reduction of the average startle amplitude (A):

(1) $$\begin{array}{cc} \begin{array}{l} \text{PPI}[\%]=\left({\text{A}}_{\text{startle alone}}-{\text{A}}_{\text{prepulse+startle}}\right)\\ \;\times 100/{\text{A}}_{\text{startle alone}} \end{array} & \left(1\right) \end{array}$$

Startle events were defined as A_startle alone_ > 30mV or A_prepulse + startle_ with PPI <15%. PPI events were defined as A_prepulse + startle_ with PPI >15% (Geyer and Swerdlow, 1998). The number of startle and PPI events was determined for each animal, and the relative difference was calculated:

(2) $$\begin{array}{cc} \text{Rel}.\text{diff}.[\%]=\left({\text{n}}_{\text{PPI}}-{\text{n}}_{\text{startle}}\right)\times 100/\left({\text{n}}_{\text{PPI}}+{\text{n}}_{\text{startle}}\right) & \left(2\right) \end{array}$$

Negative values indicate more startle whereas positive values signify more PPI incidents.

#### Startle control

The startle control session resembled the PPI session, except that all paired prepulse-startle trials were substituted by either startle-alone or background noise trials. The total number of startle trials was individually set to the number of startle reactions this animal had displayed in the PPI paradigm (range: 65–127).

#### Background noise control

For background control, rats were placed for 45 min into the SR-Lab, but were exposed solely to continuous background noise (65 dB SPL LIN).

### Behavioral PET imaging

Behavioral PET experiments were accomplished on separate days whereby animals started in a counterbalanced order either with the background control or PPI paradigm. The startle control always followed the PPI test. Each animal received an i. p. injection of 500–700 μl [^18^F]fluoro-2-deoxyglucose solution (FDG, ~2 mCi). After three minutes, rats underwent one of the conditions described above for 45 min, during which FDG accumulated in energy-consuming brain cells. Afterwards, rats were anesthetized [initial dosage: 5% isoflurane in O_2_/N_2_O (3:7)], placed on an animal holder (medres®, Cologne, Germany), and fixed with a tooth bar in a respiratory mask. Static PET scans in list mode were performed using a Focus 220 micro PET scanner (CTI-Siemens®) with a resolution at center of field of view of 1.4 mm. Data acquisition started exactly 1 h after FDG-injection and lasted 30 min. Breathing rate was monitored and kept around 55/min by adjusting isoflurane concentration (1.5–2.5%). Body temperature was maintained at 37°C by a feedback-controlled system. Following Fourier rebinning, data were reconstructed using the iterative OSEM3D/MAP procedure (Qi et al., 1998) resulting in voxel sizes of 0.38 × 0.38 × 0.82 mm.

### Magnetic resonance imaging (MRI)

MRI scans were performed in an 11.7-T BioSpec animal scanner (Bruker BioSpin®, Billerica, MA, USA) using a quadrature receive-only rat brain surface coil (Bruker BioSpin®) in combination with an actively decoupled, transmit-only quadrature resonator with 72 mm inner diameter (Bruker BioSpin®), fitting into the BFG-150/90-S14 combined gradient and shim set of 90 mm inner diameter (Resonance Research Inc., Billerica, MA, USA) with a maximum gradient strength of 745 mT/m. A T2-weighted sequence, rapid acquisition with relaxation enhancement (RARE) was used: RARE factor = 8, repetition time/effective echo time = 6500/32.5 ms, averages = 2, matrix size = 256 × 256, FOV = 3.2 × 3.2 cm^2^, 58 slices, slice thickness = 0.5 mm, interslice spacing = 0.5 mm. Inhalation anesthesia procedures were similar to those used during PET scans.

### Data analysis

Imaging data were analyzed using the imaging software tool VINCI 4.04 (Vollmar et al., 2007). MR images were used to screen for gross structural anomalies, e.g., ventricular enlargement. From initially 22 rats scheduled for the PET study, three had to be excluded due to structural non-conformity. Furthermore, MR images were involved in the coregistration procedure and served as anatomical templates for projection of statistical PET maps. For intensity normalization of PET images, the olfactory bulb was chosen as reference area. Olfactory activity was supposed to be similar in both conditions, i.e., rats were exposed to the smell of diluted acetic acid. According to the ratio normalization technique (Arndt et al., 1996), each PET image was divided by the value of the respective olfactory bulb volume of interest (VOI). Images (Figure 2A) were not further pre-processed, e.g., no spatial normalization or Gauss filtering was done. Statistical analysis of imaging data was performed in VINCI using a Python-based statistics tool. Metabolic differences between PPI paradigm, startle and background control were assessed with One-Way repeated measures ANOVA followed by Tukey *post-hoc* multiple comparison (*n* = 9 Black hooded rats). In addition, images from the PPI paradigm were compared to background control using a paired *t*-test (*n* = 14 Black hooded rats). For correlative analysis (14 Black hooded and 5 Lister hooded rats), the Pearson product-moment correlation test was used to assess the relationship between difference images (PPI paradigm minus background) and the relative difference of PPI and startle events (Equation 2).

In order to estimate statistical noise in our PET data, we used two different procedures: (1) Background control images of the 14 Black hooded rats were randomly divided into two groups of seven animals. The two groups were compared using a two-sample *t*-test, and mean *t*-value and standard deviation were calculated. (2) Left and right hemispheres of the 14 Black hooded background control images were compared. For that purpose, all images were flipped and coregistered with their original counterparts. For seven animals the right hemisphere was subtracted from the left, and for the other seven animals vice versa. These differences were used to perform a paired *t*-test. Mean *t*-value and standard deviation was calculated from the left half of the brain. *T*-values resulting from comparison of different conditions were considered above noise when they were two standard deviations higher than mean *t*-values of background control comparisons described above.

To detect symmetric patterns, a VOI analysis was performed using anatomically defined VOIs from brain areas where significant voxels were found. VOI mean values were determined to ensure that areas contralateral from clusters of significant voxels displayed metabolic changes of the same direction (increase or decrease).

For statistical analysis of behavioral data, all percentage values were arcsin-transformed, and subsequent calculations were done using SPSS-Statistics 20 (IBM®). Hearing thresholds of both rat strains were compared by a *t*-test. Relative differences of PPI and startle events as well as comparison of startle amplitudes between strains were assessed using Mann-Whitney *U*-tests. Startle amplitudes during PPI paradigm and startle control (9 Black hooded rats) were compared by a paired *t*-test. Pearson correlation analysis was used to examine the relationship between hearing threshold and relative difference of PPI and startle events. Effects of rat strain and prepulse intensity on % PPI (Equation 1) were analyzed using Two-Way mixed design ANOVA (factor 1: prepulse intensity, factor 2: strain) followed by *post-hoc* test Sidak.

In order to estimate how learning or habituation effects might contribute to metabolic differences between conditions, PPI sessions and startle control sessions were divided into an early and a late half. For each animal, an early and late mean value for startle amplitude and % PPI was calculated, and both were compared with a paired *t*-test for each rat strain.

## Results

### Hearing test

Black hooded rats (*n* = 14) had a hearing threshold of 23.6 ± 5.7 dB SPL, whereas in Lister hooded rats (*n* = 5) hearing threshold was 60.8 ± 2.4 dB SPL. This difference was statistically significant (*p* < 0.001).

### Behavior

**Figure 1:**
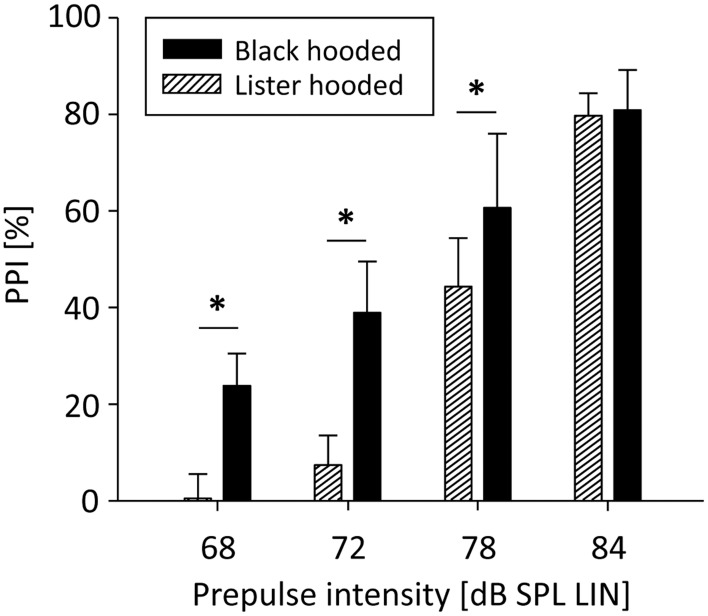
**PPI depends on prepulse intensity and hearing capacity.** PPI of Lister and Black hooded rats was a function of prepulse intensity. For low prepulses, PPI was significantly stronger in well-hearing Black hooded rats compared to hearing-impaired Lister hooded rats. For the loudest prepulse, PPI was the same in both strains (^*^p ≤ 0.001).

PPI was significantly influenced by prepulse intensity [*F*_(3, 51)_ = 342.32, *p* < 0.001]: The louder the prepulse, the stronger PPI (Figure 1). There was also a significant main effect of rat strain [*F*_(1, 51)_ = 13.26, *p* = 0.002] and a significant interaction between rat strain and prepulse intensity [*F*_(3, 51)_ = 9.93, *p* < 0.001]: For low prepulses, PPI was significantly stronger in well-hearing Black hooded rats compared to hearing-impaired Lister hooded rats, while PPI of the loudest prepulse was the same in both strains. The relative difference between PPI and startle events was significantly lower in Lister hooded (−9.0 ± 8.9%) compared to Black hooded rats (9.7 ± 13.6%; Mann-Whitney *U* = 7.0, *p* = 0.010), i.e., hearing impaired Lister hooded rats startled more often. Interestingly, the startle amplitude of Lister hooded rats (120.8 ± 59.1 mV) was significantly lower compared to that of Black hooded rats (226.0 ± 99.3 mV, mean ± SD, Mann-Whitney *U* = 3.0, *p* = 0.003). Separate analysis of the startle amplitude during the PPI paradigm and startle control condition (9 Black hooded rats) revealed a significantly lower amplitude during PPI test condition (256.5 ± 109.7 mV, mean ± SD) compared to startle control (698.2 ± 324.8 mV; *t*_(8)_ = −4.89, *p* = 0.001).

Comparisons of startle amplitudes and PPI performance from the first and second half of the PPI session showed that PPI was 4.1% lower in the late half compared to the early half of the PPI session (*p* = 0.047) in Black hooded rats. Startle amplitudes did not differ significantly, but were by trend higher in the late half of the PPI session. In the startle control session, startle amplitudes were significantly higher in the late half (by 31.8 ± 37.1%; *t*_(9)_ = −2.98; *p* = 0.018). However, the increase of startle amplitude was not correlated with the number of startle trials in the session (*R* = 0.43, *p* = 0.252). These results suggest that in both PPI and startle control sessions sensitization took place which was particularly pronounced in the startle control session, but not linked to the absolute number of startle events in a linear fashion.

### Behavioral PET imaging

#### Noise in PET data

Comparing background control images of two groups of seven randomly assigned Black hooded rats yielded a mean *t*-value of 0.22 ± 0.77 (mean ± SD). When left and right hemispheres were compared, mean *t*-value was 0.08 ± 1.10. The former was 2.5, the latter 1.9 standard deviations lower than the critical *t*-value used in the corresponding test comparing PPI and background control, which was 2.18. Therefore, the presented *t*-maps reflect metabolic changes above noise level. Furthermore, all significant clusters of voxels were accompanied by non-significant changes in the same direction (increase or decrease) in the contralateral brain area, unless otherwise indicated by adding “left” or “right.”

#### Metabolic activity during the PPI-PET session

**Figure 2:**
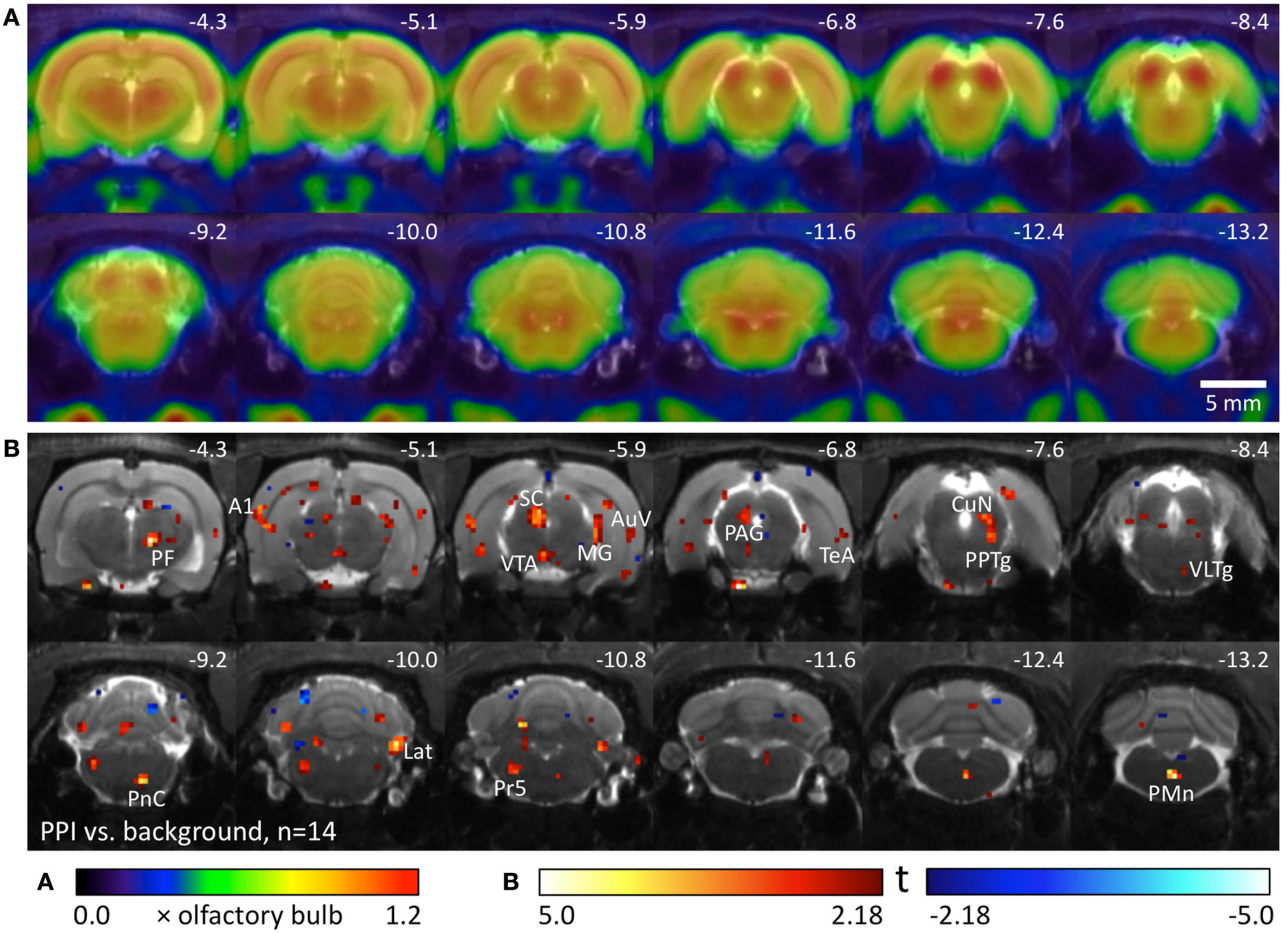
**Comparison of PPI session and background noise in well-hearing Black hooded rats. (A)** Average FDG images derived from 9 awake Black hooded rats exposed to background noise, projected on an MR image. PET image intensity was normalized to olfactory bulb. **(B)** T-map computed from a paired *t*-test comparing images from the PPI-PET session with those from background noise control. Red color indicates voxels with higher FDG uptake in the PPI-PET session. Blue color indicates voxels with higher FDG uptake during background noise. Only voxels with *p*-values <0.05 (uncorrected) are shown. Numbers represent stereotactic rostrocaudal coordinates in mm with respect to Bregma. A1, primary auditory cortex; AuV, secondary auditory cortex, ventral area; CuN, cuneiform nucleus; Lat, lateral (dentate) cerebellar nucleus; MG, medial geniculate nucleus; PAG, periaqueductal gray; PF, parafascicular thalamic nucleus; PMn, paramedian reticular nucleus; PnC, caudal pontine reticular nucleus; PPTg, pedunculopontine tegmental nucleus; Pr5, principal sensory trigeminal nucleus; SC, superior colliculus; TeA, temporal association cortex; VLTg, ventrolateral tegmental area; VTA, ventral tegmental area.

We used 14 well-hearing Black hooded rats to compare metabolic brain activity during the PPI test with activity during background noise. Metabolic activity during PPI-PET (Figure 2) was elevated in the main area of the startle pathway, the caudal pontine reticular nucleus (PnC), as well as in areas of the PPI mediation pathway: ventral inferior colliculus (IC), left superior colliculus (SC), and pedunculopontine tegmental nucleus (PPTg). In the telencephalic auditory system, metabolic activity was increased in the medial geniculate (MG), primary auditory cortex (A1), ventral secondary auditory cortex (AuV), and temporal association cortex (TeA). Furthermore, enhanced activity was found in the right parafascicular thalamic nucleus (PF), medial part of the ventral tegmental area (VTA), left periaqueductal gray (PAG), cuneiform nucleus (CuN), right lateral cerebellar nucleus (Lat), principal sensory trigeminal nucleus (PrV), and paramedian reticular nucleus (PMn).

#### Startle control condition

**Figure 3:**
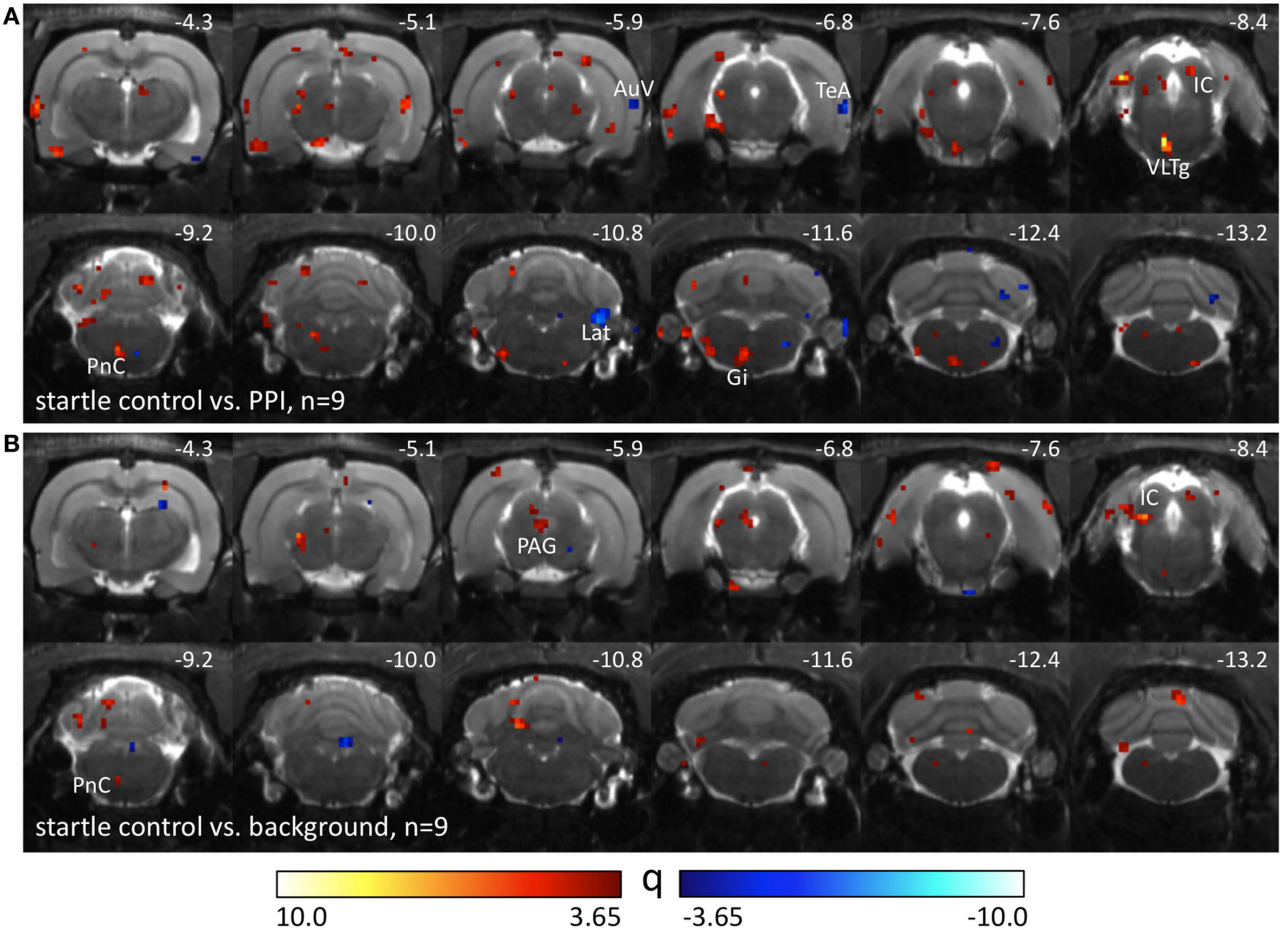
**Comparison of different PET sessions in well-hearing Black hooded rats.** Q-maps resulting from Tukey *post-hoc* tests following a One-Way repeated measures ANOVA which compare images from the startle control PET session with those from **(A)** PPI and **(B)** background control sessions. Red color indicates voxels with higher FDG uptake in the startle control PET session. Blue colored voxels represent higher FDG uptake during the PPI paradigm or background control. AuV, secondary auditory cortex, ventral part; Gi, gigantocellular reticular nucleus; IC, inferior colliculus; Lat, lateral (dentate) cerebellar nucleus; PAG, periaqueductal gray; PnC, caudal pontine reticular nucleus; TeA, temporal association cortex; VLTg, ventrolateral tegmental area.

Nine Black hooded rats underwent another control condition without pairings of prepulses and startle stimuli. The number of startle trials was individually adjusted to the number of startle events the animals had displayed in the previous PPI-PET session. Therefore, differences between startle control and PPI condition (Figure 3A) were assumed to be caused by prepulse-related processing. Elevated metabolic activity during startle control compared to the PPI session presumably reflects a startle-related synaptic input which is suppressed during PPI, particularly if it is visible in the comparison between startle and background control as well (Figure 3B). This comprises not only sensorimotor processing of startle stimuli, but also sensitization effects leading to an increase of startle amplitudes during the session. Here we identified the left PnC, left VLTg, left gigantocellular reticular nucleus (Gi), and IC. The PAG seemed to be equally involved in startle and PPI. Decreased metabolic activity during startle control indicates PPI-related synaptic activity, which was found in the right AuV, right TeA, and right Lat.

#### Correlative analysis

**Figure 4:**
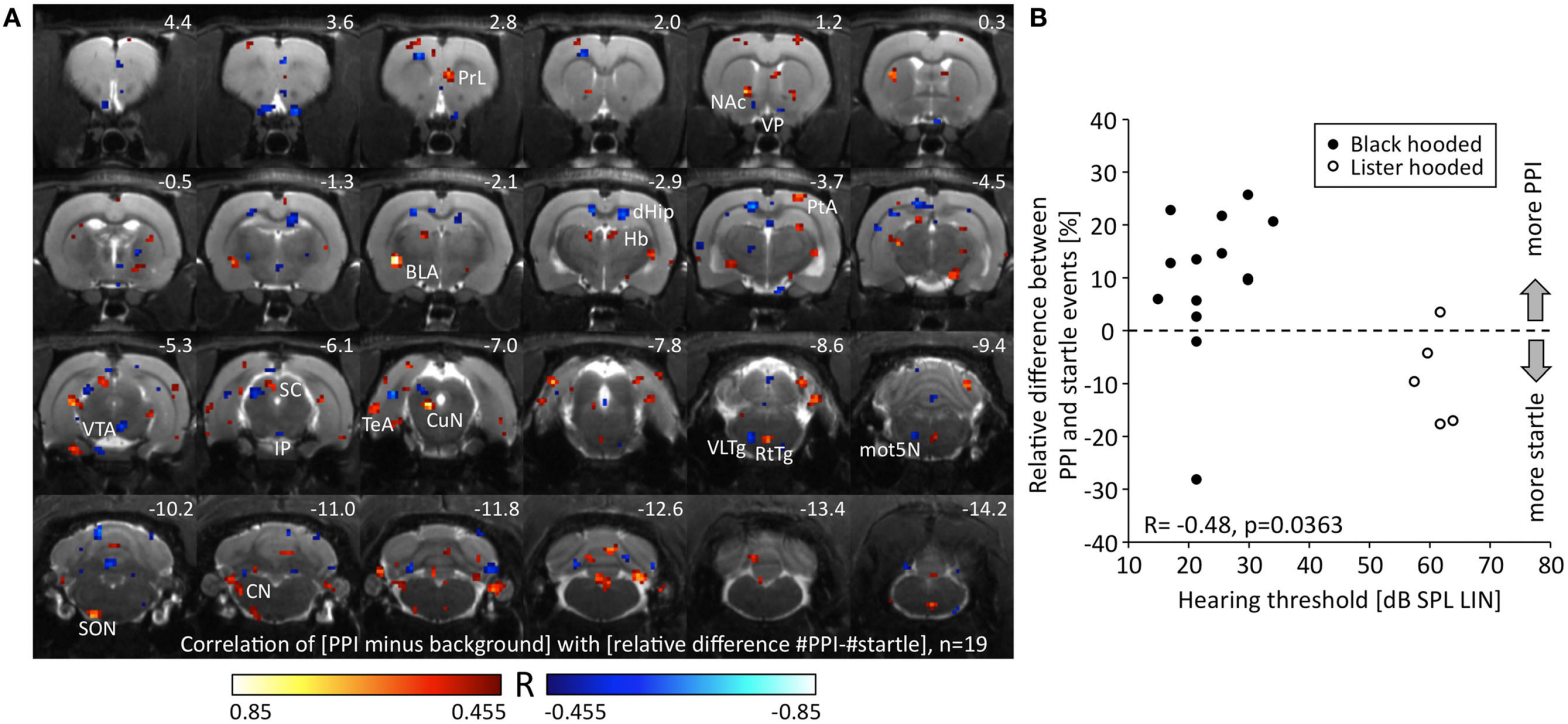
**Relationship between metabolic activity and PPI/startle. (A)** R-map resulting from a Pearson correlation test linking the change of metabolic activity during PPI-PET vs. background with the relative difference between PPI and startle events. Red colored voxels depict positive correlation, i.e., the more PPI events, the higher the metabolic activity. Blue color indicates voxels with negative correlation, i.e., the more startle events, the higher the metabolic activity. Only voxels with a significant *R*-value are shown (*p* < 0.05, uncorrected). **(B)** The relative difference between PPI and startle events was negatively correlated to hearing threshold. The better the hearing capacity the higher was the relative number of PPI events. Note that a wide range of PPI/startle events is covered by the collective of animals. The relative difference of events was significantly lower in Lister hooded compared to Black hooded rats (Mann-Whitney *U* = 7.0, *p* = 0.010). BLA, basolateral amygdala; CN, cochlear nucleus; CuN, cuneiform nucleus; dHip, dorsal hippocampus; Hb, habenula; IP, interpeduncular nucleus; mot5N, motor trigeminal nucleus; NAc, nucleus accumbens; PrL, prelimbic cortex; PtA, parietal association cortex; RtTg, reticulotegmental nucleus of the pons; SC, superior colliculus; SON, superior olivary nucleus; TeA, temporal association cortex; VLTg, ventrolateral tegmental area; VP, ventral pallidum; VTA, ventral tegmental area.

We correlated the change of metabolic activity during PPI test condition compared to background control with the relative difference between PPI and startle events. Since the relative difference between PPI and startle events was negatively correlated to hearing threshold (*R* = −0.48, *p* = 0.036, Figure 4B), we also included the five hearing-impaired Lister hooded rats to obtain a wider range of behavioral data (see above). A negative correlation between metabolic activity change and relative difference of events identified voxels (Figure 4A; blue), which were associated with the relative number of startle events. They were located in the area of the ventral pallidum (VP), dorsal hippocampus (dHip), right VTA, interpeduncular nucleus (IP), deep layers of the left SC (dlSC), ventrolateral tegmental area (VLTg), and left motor nucleus of the trigeminal nerve (mot5N). A positive correlation indicated voxels (Figure 4A; red), where metabolic activity was related to the relative number of PPI events and hence to PPI processing. They were found in the right prelimbic cortex (PrL), core of nucleus accumbens (NAc), left basolateral amygdala (BLA), habenula (Hb), right parietal association cortex (PtA), left CuN, reticulotegmental nucleus (RtTg), left superior olivary nucleus (SON), and left cochlear nucleus (CN).

## Discussion

The aim of our study was to functionally validate proposed neural networks of startle and PPI processing in intact, awake animals. FDG-PET has the advantage compared to other imaging modalities that the actual trapping of the tracer occurs during a behavioral task, whereas the trapped tracer can be imaged thereafter under anesthesia. This requires well-designed control measurements in order to interpret the results precisely. We have to keep in mind that the most energy-consuming process is the activity of dendritic and astrocytic ion pumps, which is closely linked to transmitter release (Adams and Martin, 1996; Raichle and Mintun, 2006; Figley and Stroman, 2011). Thus, FDG uptake corresponds mainly to afferent activity, whereby activations represent elevated input activity of both excitatory and inhibitory type (Ackermann et al., 1984; Nudo and Masterton, 1986; Ritter and Villringer, 2002). Deactivations indicate reduced input activity, except for areas with high spontaneous activity, e.g., VP around 9 Hz (Chrobak and Napier, 1993). Here, deactivations most likely denote a decrease of spontaneous activity, caused by inhibitory input.

In order to obtain a wider spectrum of behavior for correlative analysis, we used two genetically related rat strains, Black hooded and Lister hooded rats, with different hearing abilities. BAEP-measurements revealed that the hearing acuity of Black hooded rats was similar to other rat strains (Backoff and Caspary, 1994; Brandt-Lassen et al., 2000; Popelar et al., 2006), whereas the hearing threshold of Lister hooded rats was elevated by 40 dB. In both strains, PPI was a function of prepulse intensity, which is a basic property of this behavior (Hoffman and Wible, 1970; Ison, 1978; Reijmers and Peeters, 1994). This indicates that the general ability for PPI was intact in hearing-impaired rats.

Although all prepulse and startle pulse intensities exceeded hearing threshold of both strains, different hearing abilities were reflected in both startle amplitude and magnitude of PPI as well as the actual number of startle and PPI events. In both strains, PPI was significantly dependent on prepulse intensity, which corresponds to literature (Hoffman and Wible, 1970; Ison, 1978; Reijmers and Peeters, 1994). However, Lister hooded rats exhibited lower startle amplitude, but startled more often than Black hooded rats, because they perceived the startle stimulus not that loud and low amplitude prepulses did not induce PPI. This indicates that PPI depends on prepulse salience against background noise. Human studies further circumstantiated that the signal-to-noise ratio is more important than absolute prepulse intensities for the magnitude of PPI (Franklin et al., 2007). With the experimental setup used, a signal-to-noise ratio of 1.3 was high enough to evoke maximal PPI in both strains, while PPI differed notably with signal-to-noise-ratios of 1.04–1.11 between Black and Lister hooded rats. It should therefore be considered that disagreement about PPI magnitudes throughout the literature might be caused by different hearing acuities rather than by PPI-related genetic strain differences.

In order to analyze the neural network related to startle and PPI, we included solely well-hearing Black hooded rats in the subtractive, and all rats in the correlative analysis.

### Startle pathway

Our results confirmed the importance of the PnC as part of the startle pathway, together with the more rostrally located VLTg. VLTg activity was correlated to the relative number of startle events, and both areas were activated in the PPI paradigm vs. background as well as in the startle control vs. PPI. The latter indicates that in the startle control reticular and tegmental input activity was higher than in the PPI paradigm, presumably owing to higher mean startle amplitudes and the ascending startle amplitudes over time. We also confirmed the motN5 as a motor element of the startle pathway, since it was activated during the PPI paradigm and appeared as startle-related in the correlative analysis.

### PPI mediation pathway

Areas of the PPI mediation pathway should be visible during the PPI paradigm vs. background, but not necessarily in the difference image of startle control and PPI. If we assume that every acoustic stimulus activates the PPI mediation pathway, and only the timing of converging PnC/VLTg inputs from the CN (excitatory) and PPTg (inhibitory) determines PPI, the identical acoustic stimulation energy in both conditions will result in identical metabolic activation patterns. In the PPI paradigm vs. background, ventral IC, SC, and PPTg were activated, which is in line with classical PPI mediation pathway. In addition, the CuN was activated and its metabolic activity was correlated to the relative number of PPI events. It has been shown that mimicking prepulses by electrical CuN stimulation evokes PPI (Saitoh et al., 1987), while electrolytic lesions of the lateral tegmental area including CuN reduce PPI (Leitner et al., 1981) and increase startle amplitude in startle-alone trials (Swerdlow and Geyer, 1993). Since the CuN projects heavily to PnC and other nuclei of the medullar reticular formation (Korte et al., 1992), the CuN most likely is another area involved in PPI mediation.

### PPI modulation network

**Figure 5:**
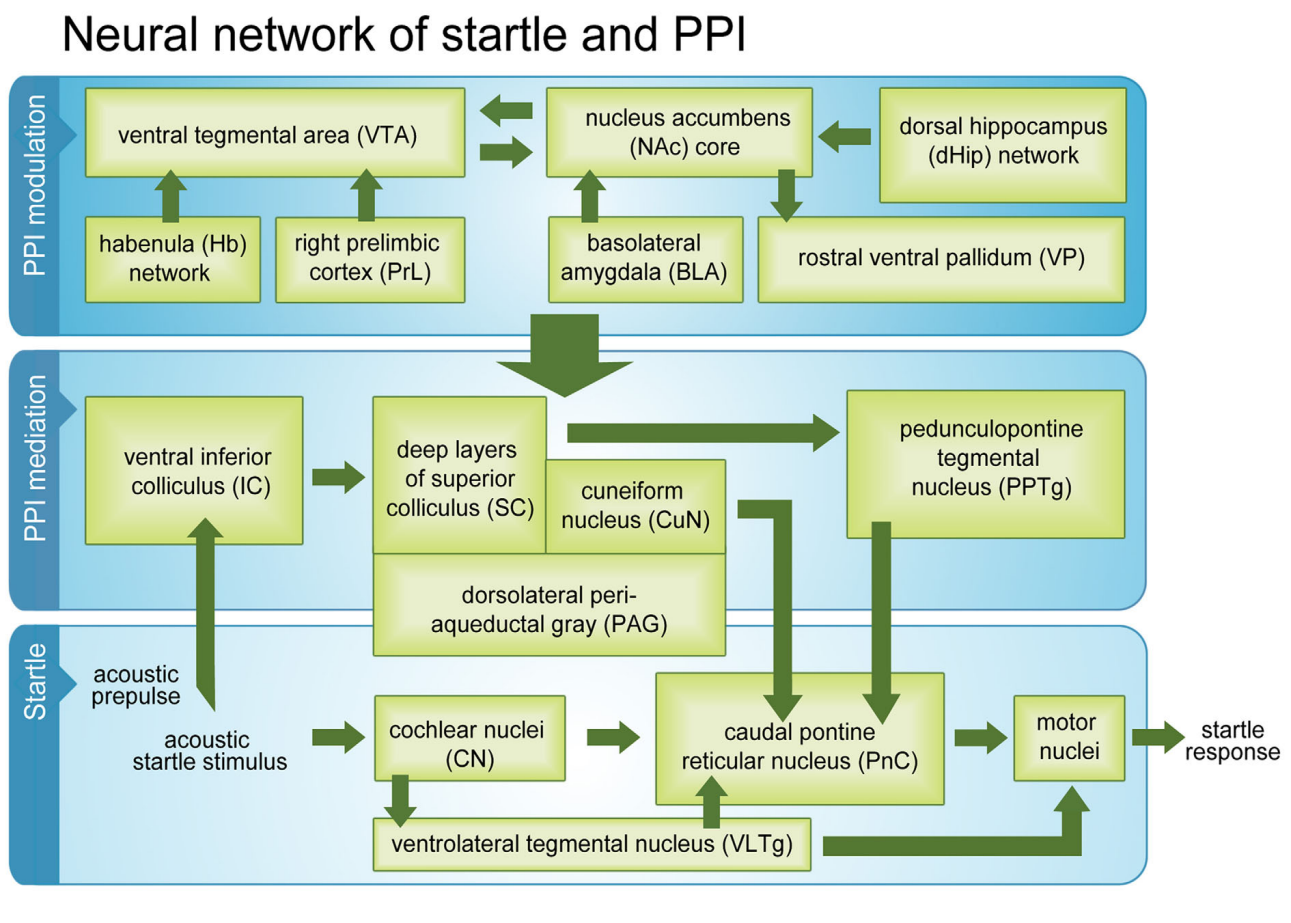
**Startle and PPI mediation pathways and PPI modulation network.** Activated brain regions (green boxes) during a passive PPI session in intact rats. Beside classical startle and PPI mediation networks, the modulation network was fully activated.

Numerous brain areas have been included in the PPI modulation network so far, but it is still a matter of debate how it is activated in intact animals, and what functions it serves. Since the PPI modulation network mainly comprises brain regions associated with the limbic system, we can assume that it is involved in representation of emotional salience and valence of stimuli (Kraus and Canlon, 2012). It is conceivable that this network evaluates if prepulse and/or startle stimuli are potentially harmful (Filion et al., 1993), and adjusts the attentive state of the animal accordingly. The influence of attention on PPI has been extensively demonstrated using the attention-to-prepulse paradigm in humans (Li et al., 2009) and rats (Roskam and Koch, 2006). Activations in relation to the relative number of startle or PPI events may therefore represent the current filtering status within the network as well as ongoing modulation of sensorimotor gating. The PPI mediation and startle pathways might be connected with the PPI modulation network (Figure 5) via PPTg and CuN (see above), but also via the dorsolateral PAG and dlSC, which were activated in the PPI paradigm and startle control. Excitation of the dorsolateral PAG increases fear-potentiation of startle response (Fendt, 1998), and stimulation of PAG together with dlSC sensitizes rats to anxiety-like behaviors (De Almeida et al., 2006). It has been proposed that CuN, PAG, and dlSC act as a functional unit to control the activity of the ventral medulla (Zemlan and Behbehani, 1984), and our results support this hypothesis.

Within the modulation network, we identified dHip, rostral VP, and VTA as startle-related. In dHip, input activity was the higher the more often the animal startled, which might be associated with consolidation of fear memories (Fendt et al., 2005; Zelikowsky et al., 2012) influencing the long-term attentional state of the animal. In rostral VP, it is most likely that spontaneous activity had increased in relation to startle due to a reduced GABAergic input from NAc medium spiny neurons. Interestingly, the NAc core was activated in relation to PPI events. This implies a decreased NAc core activity during startle, resulting in an elevated spontaneous VP activity. VP sends GABAergic projections to PPTg (Swanson et al., 1984; Mogenson et al., 1985; Smith et al., 2009), which in turn inhibits the startle pathway areas PnC and VLTg. An increase in VP output activity might therefore disinhibit these startle-related areas, resulting in enhanced startle amplitude and attenuated PPI. The startle-potentiated startle paradigm (McQueen et al., 2001; Winston et al., 2001; Commissaris et al., 2004) revealed that anticipation of the startle stimulus is sufficient to increase startle amplitudes.

In further extension of previous models, we found that metabolic activity of the Hb was associated with PPI events. Since the right medial and left lateral Hb appeared in the correlation map, we assume that both subnuclei are involved in PPI modulation. The medial Hb sends excitatory projections to IP (Qin and Luo, 2009; Ren et al., 2011), which was negatively associated with PPI events (i.e., positively associated with startle). It is therefore conceivable that the increased medial Hb input we saw in relation to PPI was inhibitory, possibly mediated by the exceptionally large number of GABA(B) receptors found in this area (Kaupmann et al., 1998). The finding that absence of the medial Hb-IP pathway in genetically modified mice reduces PPI (Kobayashi et al., 2013) supports the contribution of medial Hb to the PPI modulation network. The lateral Hb receives input from the basal forebrain and integrates responses to aversive stimuli (Nair et al., 2013). By controlling the midbrain aminergic systems (Geisler and Trimble, 2008), it may be involved in the regulation of the animal's attentive and vigilance state. Lateral Hb activity indirectly inhibits midbrain dopaminergic neurons (Hikosaka et al., 2008), which is reflected by the negative correlation between metabolic activity in VTA and the number of PPI events in our study.

The right PrL was the only prefrontal cortical region positively associated with PPI events. It has been suggested that the ventral hippocampal (vHip)-PrL projection is involved in PPI modulation (Shoemaker et al., 2005; Kamiyama et al., 2011), but our results do not provide evidence for vHip activation. The exclusive mapping of the right PrL may reflect a unilateral function, because lesion experiments have already suggested that activation of the PrL/infralimbic (IL) region during potentially threatening situations is lateralized to the right hemisphere (Sullivan and Gratton, 2002). However, the same study provided no evidence for lateralization of PPI processing. This may be explained by the fact that lesions did not reach the most dorsal part of PrL/IL, where PPI processing takes place according to our results.

Taken together, our results confirmed almost all areas previously identified as belonging to the startle pathway, PPI mediation pathway, and PPI modulation network. However, we were able to additionally strengthen current evidence that the Hb is an integral part of the PPI modulation network, which seems to be connected to PPI mediation areas via PPTg, CuN, PAG, and dlSC. To the best of our knowledge this study is the first that demonstrated that the whole PPI modulation network is fully active during “passive” standard PPI sessions, where no selective attention to prepulse or startle stimulus is required. We conclude that activity of the PPI modulation network during passive PPI reflects ongoing monitoring of stimulus significance and continuous adjustment of sensorimotor gating. It is conceivable that activation of the PPI modulation network during the PPI session prevented sensitization of the startle response, which occurred during the startle control condition.

Furthermore, our results demonstrate that behavioral FDG-PET is a feasible method for functional network analysis in freely-moving small animals. It not only adds to the results of invasive studies, but can also be combined with lesion experiments, pharmacological treatment, or transgenic models. The possibility of longitudinal studies including several measurements in freely-moving animals is a remarkable advantage that allows various study designs comparable to human fMRI studies. The next step in investigating PPI will be to monitor network activity in rodent models of neurological and psychiatric disorders, in order to evaluate pathological network activity and treatment effects.

## Author contributions

Cathrin Rohleder, Heike Endepols, and F. Markus Leweke conceived the study. Cathrin Rohleder and Heike Endepols developed and organized the study with input from F. Markus Leweke and Rudolf Graf. Fabienne Jung, Bernd Neumaier, Dirk Wiedermann, and Michael Sué contributed reagents/materials/analysis tools. Cathrin Rohleder, Fabienne Jung, Hanna Mertgens, and Heike Endepols performed experiments. Heike Endepols and Cathrin Rohleder analyzed data and drafted the manuscript. All authors contributed to final manuscript preparation, discussed the results and their implications, and have read and approved the final manuscript.

### Conflict of interest statement

The authors declare that the research was conducted in the absence of any commercial or financial relationships that could be construed as a potential conflict of interest.
